# Altered regional homogeneity of spontaneous brain activity in patients with toothache: A resting-state functional magnetic resonance imaging study

**DOI:** 10.3389/fnins.2022.1019989

**Published:** 2022-09-28

**Authors:** Jun Yang, Yi Shao, Bin Li, Qiu-Yue Yu, Qian-Min Ge, Biao Li, Yi-Cong Pan, Rong-Bin Liang, Shi-Nan Wu, Qiu-Yu Li, Yu-Lin He

**Affiliations:** ^1^The Key Laboratory of Oral Biomedicine, The Affiliated Stomatological Hospital of Nanchang University, Nanchang, China; ^2^Department of Ophthalmology, The First Affiliated Hospital of Nanchang University, Nanchang, China; ^3^Department of Radiology, The First Affiliated Hospital of Nanchang University, Nanchang, China

**Keywords:** toothache, regional homogeneity, rs-fMRI, neuroimaging, disease markers

## Abstract

Toothache (TA) is a common and severe pain, but its effects on the brain are somewhat unclear. In this study, functional magnetic resonance imaging (fMRI) was used to compare regional homogeneity (ReHo) between TA patients and a normal control group and to explore the brain activity changes during TA, establishing the theoretical basis for the mechanism of neuropathic pain. In total, 20 TA patients and 20 healthy controls (HCs) were recruited and underwent assessment of pain, and then resting-state fMRI (rs-fMRI). The ReHo method was used to analyze the original whole-brain images. Pearson’s correlation analysis was used to assess the relationship between mean ReHo values in each brain region and clinical symptoms, and the receiver operating characteristic (ROC) curve was used to conduct correlation analysis on the brain regions studied. The ReHo values of the right lingual gyrus (RLG), right superior occipital gyrus (RSOG), left middle occipital gyrus (LMOG) and right postcentral gyrus (RPG) in the TA group were significantly higher than in HCs. The mean ReHo values in the RLG were positively correlated with the anxiety score (AS) (*r* = 0.723, *p* < 0.001), depression score (DS) (*r* = 0.850, *p* < 0.001) and visual analogue score (VAS) (*r* = 0.837, *p* < 0.001). The mean ReHo values of RSOG were also positively correlated with AS (*r* = 0.687, *p* = 0.001), DS (*r* = 0.661, *p* = 0.002) and VAS (*r* = 0.712, *p* < 0.001). The areas under the ROC curve of specific brain area ReHo values were as follows: RLG, 0.975; RSOG, 0.959; LMOG, 0.975; RPG, 1.000. Various degrees of brain activity changes reflected by ReHo values in different areas of the brain indicate the impact of TA on brain function. These findings may reveal related neural mechanisms underlying TA.

## Introduction

Toothache (TA) is the most common and severe pain in the field of stomatology. The oral area is distinct from other parts of the body due to its special anatomical characteristics and receptors. Medical images of typical TA show that it is often accompanied by swollen gums and severe alveolar bone resorption. TA is a symptom of potential damage, such as pulp infection. Clinical treatment to alleviate pain mainly targets pathological changes and is based on the assumption: the pain sensation of peripheral innervation can often be accurate positioning. Studies have shown that the treatment of tooth lesions does not achieve the purpose of pain relief. The reason was that the root cause of TA in some TA patients was not the teeth themselves, but the pain in this area caused by trigeminal neuropathy and bone afferent pain (Nixdorf et al., 2012). In addition, the degree of TA and the severity of lesions do not show a linear relationship, and the pain experienced is instead closely related to personal characteristics, psychological factors, and other factors (Wiech et al., 2008). Therefore, it is important to understand the cognitive regulation of the pain system to explore brain function in TA.

Resting-state functional magnetic resonance imaging (rs-fMRI) is a functional brain imaging technique that records signals reflecting metabolic changes related to neural activity in specific brain regions. The consistency of blood oxygen level-dependent (BOLD) signals between adjacent voxels in the brain is measured to evaluate the brain activity of subjects at rest (Zang et al., 2004; Biswal, 2012; Lin et al., 2013a). At each location in the brain, neighboring voxel BOLD signals show similar modulation over time. The ReHo is calculated using the REST toolkit using Kendall’s coefficient of consistency (KCC) (Kippenhan et al., 1992). The ReHo (rs-ReHo) diagram of an individual’s resting state is generated by combining the KCC value of each voxel and its 26 nearest neighboring voxels (Zang et al., 2004). Individual rs-ReHo diagrams are standardized with their own average KCC. Based on this concept, the regional homogeneity (ReHo) method can be used to measure the degree of similarity of different voxel time series in brain regions, to obtain information about brain activity, and determine the spontaneous fMRI signal partial synchronization (Brügger et al., 2011, 2012). As one of the data-driven methods for the study of fMRI, ReHo can collect more regional functional activity information than the model-driven method without any prior assumptions about the hemodynamic model, to further analyze the functional activity changes of temporary specific brain regions performing corresponding tasks (Zang et al., 2004). One of the advantages of ReHo is that it does not require random seed assignment and can be used to detect changes in any region of the cerebral (Tononi et al., 1998). In addition, compared with other traditional ALFF sequences, ReHo sequences can observe synchronous signals of neural functional activity in cerebral regions with similar functional activity changes. Therefore, the sequence can be used to analyze the corresponding cerebral regions as a whole and expand the wholeness of observation (Yue et al., 2020). Up to now, ReHo has also been used by some researchers in the study of pathology in psychiatric, neurological, and ophthalmic diseases such as the pathogenesis of epilepsy (Tang et al., 2014), optic neuritis (Shao et al., 2015), and comitant strabismus (Huang et al., 2016b).

Neuro-electrophysiology and neuro-orbital procedures have shown that structural networks involved in the multidimensional aspects of pain perception mainly included the primary and secondary somatosensory cortex, insula cortex, the anterior cingulate, prefrontal cortex, the thalamus, and cerebellar cortex (Apkarian et al., 2005; Farrell et al., 2005). Studies have found that the influence of TA on brain-associated networks is strikingly similar to that reported for spinal pain (Iannetti and Mouraux, 2010). A previous study reported the changes in brain functional connections in TA and found that the functional activities of the right lingual gyrus, right precentral gyrus, and left middle temporal gyrus were significantly higher than those of healthy subjects (Wu et al., 2020). The study also first confirmed that the degree centrality (DC) value of the right gyrus was positively correlated with the anxiety and depression scores of the subjects, and the DC value of the left middle temporal gyrus was positively correlated with the pain score. Our other previous study using ALFF sequence to study the changes in brain functional activities in TA patients also showed similar results (Yang et al., 2019). Studies on the changes in functional activities in the corresponding brain regions of TA showed that different levels of TA stimulation were closely related to specific subregions in the cerebral cortex pain network, including the bilateral anterior insula and cingulate gyrus (Brügger et al., 2012). However, these pain-related brain changes which had been confirmed maybe not be caused by pain entirely, even if the location of the lesions and the nature of the stimulus are the same. The study of the changes in cerebral functional activity in patients with TA provides conditions for further study of the neuropathological mechanism of TA. To a certain extent, the severity of the disease and the exposure to clinical symptoms can be evaluated by monitoring the changes in cerebral function and activity, to provide personalized treatment for patients with TA. The purpose of this study was to analyze the relationship between changes in ReHo values alternations in specific brain regions and clinical outcomes in TA patients by processing fMRI data using the ReHo method.

## Materials and methods

### Participants

A total of 20 patients with TA and 20 healthy controls (HCs) were recruited at the first affiliated Hospital of Nanchang University. The inclusion criteria of TA patients were: (1) pulp and/or periodontal pain due to dental or non-dental disease; (2) acute and/or chronic TA (including trigeminal nerve pain); (3) no other comorbid pain diseases; (4) no contraindications to MRI examination; (5) no obvious abnormal signals showed in Routine MRI T1WI and T2WI sequences. Exclusion criteria included: (1) structural brain abnormalities, (2) systemic disease, recent intake of analgesic drugs, anti-inflammatory or narcotic drug allergy history, (3) long-term anxiety, depression, and other mental health abnormalities, (4) heart pacemakers and other metals in the body, contraindicating an MRI scan. A visual analog scale (VAS) was used to evaluate subjects’ pain (Bijur et al., 2001). A score of 0 represented painlessness, and a score of 10 denoted excruciating pain. In addition, all subjects completed the Hospital Anxiety and fepression Scale (HADS) (Bjelland et al., 2002). Subjects were instructed to take no alcohol for 12 h before testing and MRI measurements were performed between 1 PM and 9 PM.

This study met the requirements of the Medical Ethics Committee of the first affiliated Hospital of Nanchang University. The approval number is cdyfy2017021. Subjects participating in this study expressed their willingness to participate and signed a declaration of informed consent after learning about the purpose, methods, and potential risks of the study.

### Magnetic resonance imaging parameters

The Trio 3T MR scanner (Siemens Healthcare, Erlangen, Germany) was used for MRI scans Whole-brain T1 images were weighted using prepared magnetic gradient-echo images (MPRAGE). The corresponding metamorphic gradient echo sequence was conducted using the following parameters: repetition time (TR) 1,900 ms, echo time TE 2.26 ms, thickness 1.0 mm, gap 0.5 mm, acquisition matrix 256 × 256, the field of view (FOV) 250 mm × 250 mm and turning angle 9°. Functional images were then obtained using the gradient echo plane image sequence in the static scanning session with parameters TR 2,000 ms, TE 30 ms, thickness 4.0 mm, gap 1.2 mm, acquisition matrix 64 × 64, FOV 220 mm × 220 mm, turning angle 90° and 29 axial slices. In addition, all TA patients did not use other analgesic drugs during the rS-FMRI examination.

### Functional magnetic resonance imaging data processing

To analyze the functional images we first used MRIcro software to filter unusable data and then used fMRI data processing assistant and Statistical Parameter Mapping (SPM8) for data processing (Huang et al., 2016a). To ensure that the measurement signals from each individual were balanced, the first ten volumes recorded from each individual were deleted. A series of recent studies have shown that head movement can significantly affect the results of rs-fMRI. In this study, we used the Friston 24-head motion parameter model to reduce the potential impact of head movement on the results (Van Dijk et al., 2012; Zeng et al., 2014). In addition, we used linear regression to eliminate the effects of respiration, local small motion, and low-frequency drift on the variables from variation or movement effects. After the correction of head movement, the functional magnetic resonance image was normalized to Montreal Neurological Institute space (Tate et al., 2014). Then the images were de-trended by bandpass filtering (0.01–0.08 Hz) to reduce the influence of factors such as physiological high-frequency breathing, low-frequency drift, and cardiac noise. Through the above data preprocessing methods, we ensure the image quality included in the experiment. Voxel calculations were then made in 27 individual clusters using the KCC. Based on the ReHo method, KCC was used to calculate the similarity between the time series of a given voxel and its nearest time series at the voxel level, to judge the functional activity changes of the corresponding cerebral regions. The details of the KCC calculation can be found in the references (Zang et al., 2004). The remaining data were smoothed using a full width half maximum Gaussian of 6 mm × 6 mm × 6 mm.

### Regional homogeneity statistical analysis

ReHo computations were conducted using REST software. ReHo analysis was calculated for a given time series and its neighboring voxel time series using KCC to assess the consistency and comparability of each voxel, assuming similarity with its neighbors.

### Statistical analysis

In this study, SPSS version 16.0 (IBM Corp., USA) was used to analyze clinical variables and demographics. Comparisons were made using an independent samples *t*-test, with *p* values less than 0.05 indicating statistical significance. The SPM8 toolkit was then used for general linear model analysis. To compare ReHo between the TA and HC groups, the two-sample *t*-test was used and Gaussian random field theory was applied to adjust for multiple comparisons (*P* = 0.01, 40 voxels, AlphaSim correction).

The receiver operating characteristic (ROC) curve was used to analyze the correlation between the mean ReHo values of different brain regions in each group. After obtaining the corresponding results, Pearson’s correlation analysis was used to evaluate relationships between behavioral characteristics and ReHo values in the corresponding brain regions in the TA group, with *P* < 0.05 indicating a significant difference.

## Results

### Demographics

No significant difference in age was found between the two groups (*P* = 0.692). Demographic information and pain assessment of subjects are shown in Table 1. The mean ± standard deviation of TA duration was 0.19 ± 0.07 years.

**TABLE 1:** Demographics and behavioral results of TA and HC groups.

	TA	HC	*t*-value	*P*-value
Male/female	8/12	8/12	N/A	>0.99
Age (years)	41.18 ± 11.65	42.73 ± 12.66	0.094	0.692
Handedness	18 R	18 R	N/A	>0.99
Duration (years)	0.19 ± 0.07	N/A	N/A	N/A
VAS	6.41 ± 1.69	N/A	N/A	N/A

Independent *t*-tests comparing the two groups (*p* < 0.05 represented statistically significant differences). Data shown as mean standard deviation or *n*. TA, toothache; HC, healthy control; N/A, not applicable; VAS, Visual Analogue Scale.

### Regional homogeneity differences

**FIGURE 1:**
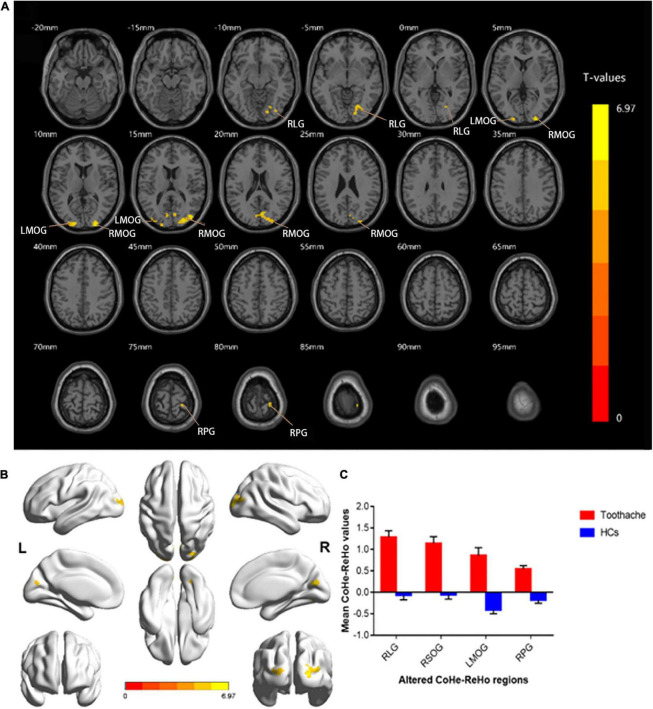
Spontaneous brain activity in the TA and HC groups. RLG, RSOG, LMOG, and RPG showed significant between-group differences in activity. Higher ReHo values are shown in red or yellow; lower values in blue [*P* < 0.01 for multiple comparisons using Gaussian random field theory (cluster 40 > voxels, AlphaSim corrected; z > 2.3, *P* < 0.01)] **(A,B)**. The mean differences in ReHo values between the HC and TA groups **(C)**. ReHo, regional homogeneity; RLG, right lingual gyrus; RSOG, right superior occipital gyrus; LMOG, left middle occipital gyrus; RPG, right postcentral gyrus; HCs, healthy controls.

ReHo values were significantly higher in patients with TA than in HCs in the following brain regions: right lingual gyrus (RLG), right postcentral gyrus (RPG), left middle occipital gyrus (LMOG), and right superior occipital gyrus (RSOG) (Figures 1A,B, yellow and Table 2). Figure 1C shows differences in mean ReHo between TA and HC groups.

**TABLE 2:** Brain regions with significant differences in CoHe-ReHo between TA patients and HC.

TA patients and HCs				MNI coordinates		
Brain areas	BA	*T*-values	Peak voxels	*x*	*y*	*z*
The right lingual gyrus	19	6.6032	43	24	–72	–3
The right superior occipital gyrus	18	6.7611	180	24	–99	6
The left middle occipital gyrus	19	6.4052	50	–18	–96	12
The right postcentral gyrus	2	6.9664	20	24	–33	81

The statistical threshold was set at voxel with *P* < 0.01 for multiple comparisons using false discovery rate. CoHe-ReHo, Coherence-based regional homogeneity; HCs, healthy controls; MNI, Montreal Neurological Institute; BA, Brodmann area.

### Correlation analysis

**FIGURE 2:**
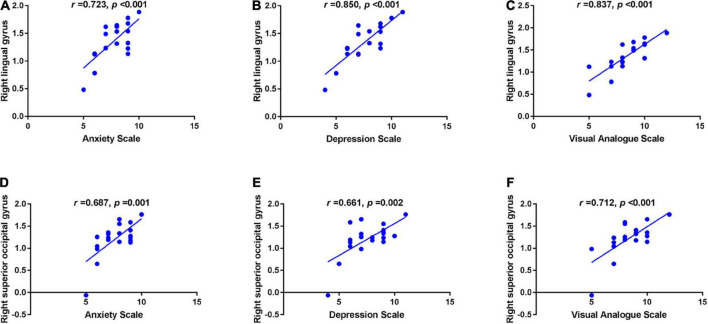
Correlations between the mean ReHo values in different brain regions and the clinical behaviors in the TA group. The mean ReHo value in the right lingual gyrus was positively correlated with the anxiety score (*r* = 0.723, *p* < 0.001) **(A)**, depression score (*r* = 0.850, *p* < 0.001) **(B)** and visual analogue score (*r* = 0.837, *p* < 0.001) **(C)**; and the right superior occipital gyrus value was positively correlated with the anxiety score (*r* = 0.687, *p* = 0.001) **(D)**, depression score (*r* = 0.661, *p* = 0.002) **(E)**, and visual analogue score (*r* = 0.712, *p* < 0.001) **(F)**. ReHo, regional homogeneity; TA, toothache; RLG, right lingual gyrus; RSOG, right superior occipital gyrus.

In the TA group, the mean ReHo values in the RLG were positively correlated with the anxiety score (AS) (*r* = 0.723, *p* < 0.001), depression score (DS) (*r* = 0.850, *p* < 0.001) and VAS (*r* = 0.837, *p* < 0.001). ReHo values at the RSOG were also positively correlated with the AS (*r* = 0.687, *p* = 0.001), DS (*r* = 0.661, *p* = 0.002) and VAS (*r* = 0.712, *p* < 0.001) (Figure 2).

### Receiver operating characteristic curve

**FIGURE 3:**
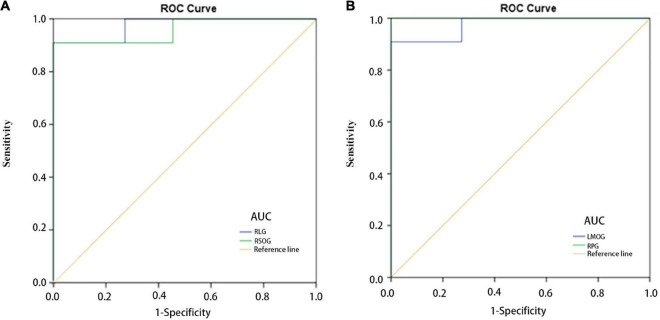
ROC curve analysis of the mean regional homogeneity values for altered brain regions. **(A)** The AUCs were 0.975 (*P* < 0.001; 95% CI: 0.918–1.000) for the RLG, and 0.959 (*P* < 0.001; 95% CI: 0.873–1.000) for the RSOG. **(B)** The AUCs were 0.975 (*P* < 0.001; 95% CI: 0.918–1.000) for the LMOG, and 1.000 (*P* < 0.001; 95% CI: 1.000–1.000) for the RPG. AUC, area under the curve; CI, confidence interval; HC, healthy control; RLG, right lingual gyrus; RSOG, right superior occipital gyrus; LMOG, left middle occipital gyrus; RPG, right postcentral gyrus; ROC, receiver operating characteristic.

We adopted the ROC curve for differential diagnosis analysis, where the area under the curve (AUC) ranges from 0.5 to 0.7, indicating that the differential diagnosis accuracy of this indicator is low; when the AUC value ranges from 0.7 to 0.9, indicating that this indicator has moderate accuracy; when the AUC value is greater than 0.9, it means excellent precision in differential diagnosis (Chen et al., 2021). Areas under the curve (AUCs) for each region were as follows: RLG, 0.975; RSOG, 0.959 (Figure 3A); LMOG, 0.975; RPG, 1.000 (Figure 3B).

## Discussion

Analysis of central nervous system activity has been an area of intense interest for many years. Since the invention of the electroencephalogram (EEG), brain function has been explored using a range of tools under various conditions. In recent decades, the characteristics of brain activity in the resting state have become a subject of great interest to various medical departments (Raichle and Snyder, 2007; Buckner et al., 2008), providing a new perspective for related studies on brain functional organization. Resting-state analysis has been used to assess ReHo values alternations relating to, movement, language, vision, processing, and other functions (Moussa et al., 2012; Lee et al., 2014). The stimuli produced by TA generate activity that may be analyzed in the resting state.

Studies have shown the importance of treating and diagnosing TA. To some extent, controlling pain is as important as treating the injury, the latter being challenging due to the difficulty in locating the damage. Even when pain-associated lesions are identified, TA is an emotional cognition-related experience. The brain affects the memory of the pain input, and related factors such as emotion and cognition can affect the patient’s experience (Tracey and Mantyh, 2007). It is difficult to study the ability of the brain to respond to non-specific memory and pain, which is largely reflected in pain caused by noxious stimuli (Mouraux et al., 2011). However, through functional methods such as fMRI, relevant information about brain pain can be obtained (Apkarian et al., 2005; Dunckley et al., 2005; Iannetti et al., 2005); many studies have used ReHo methods to explore pain-related diseases and this approach has a broad scope for further development (Table 3; Yu et al., 2012; Xie et al., 2013; Wang et al., 2014, 2015; Zhang et al., 2014; Ke et al., 2015; Wu et al., 2016; Tang et al., 2018). In addition, a study of rs-fMRI in patients with chronic cervical spondylosis showed that ReHo values in the left sensorimotor cortex and temporal lobe were significantly higher than those in normal subjects (Chen et al., 2018). This study also plays a background role in our research. ReHo has the advantage of determining the “global changes” in brain activity during pain and changes in neural activity in areas of the brain associated with pain. The pain experience is regulated by psychological, social, and other factors, with extensive inter-individual variations. Understanding how TA affects related brain mechanisms is crucial to customizing pain control for individual patients with dental disease.

**TABLE 3:** ReHo method applied in pain-related diseases.

Author	Disease	Brain regions	
		TAs > HCs	TAs < HCs
Bjelland et al. (2002)	Migraine	N/A	rACC, PFC, OFC, SMA
Huang et al. (2016a)	Idiopathic trigeminal neuralgia	ITG, thalamus, IPL, PCG	Amygdala, PHCG and cerebellum
Van Dijk et al. (2012)	Tension-type headache	N/A	CN, precuneus, putamen, MFG and SFG
Zeng et al. (2014)	Acute eye pain	SFG, IPL, precuneus	Pre/PostCG, MFG
Tate et al. (2014)	Low back pain	MPFC, precuneus, insula, PHCG, CPL	PSC, ACC, PHCG, IPL
Chen et al. (2021)	Knee osteoarthritis	Thalamus, EN, PL	Cerebrum, FL
Buckner et al. (2008)	Visceral pain	PostCG, thalamus	ACC, PFC
Raichle and Snyder (2007)	Dysmenorrhea	Declive, OFC	aIPS/SPL

ReHo, regional homogeneity; TA, toothache; HC, healthy control; N/A, not applicable; rACC, rostral anterior cingulate cortex; PFC, prefrontal cortex; OFC, orbitofrontal cortex; SMA, supplementary motor area; ITG, inferior temporal gyrus; IPL, inferior parietal lobule; PCG, postcentral gyrus; PHCG, parahippocampal gyrus; CN, caudate nucleus; MFG, middle frontal gyrus; SFG, superior frontal gyrus; Pre/PostCG, precentral/postcentral gyrus; MPFC, medial prefrontal cortex; CPL, cerebellum (posterior lobe); PSC, primary somatosensory cortex; ACC, anterior cingulate cortex; EN, extra-nucleus; PL, parietal lobe; FL, frontal lobe; PostCG, postcentral gyrus; aIPS, anterior part of the left intraparietal sulcus; SPL, superior parietal lobe.

**FIGURE 4:**
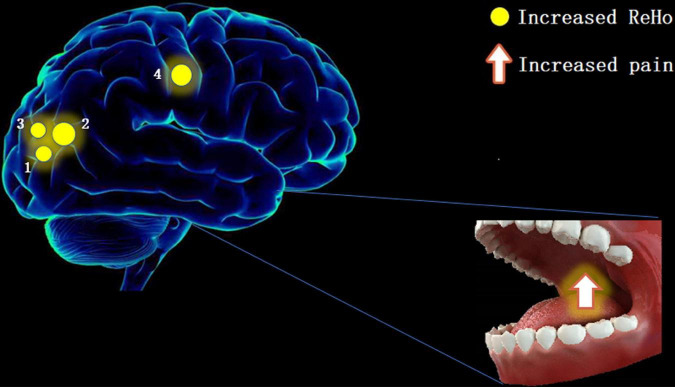
The ReHo results of brain activity in the TA group. Compared with the HCs, the ReHo value was increased in the following regions: 1—right lingual gyrus (BA19, *t* = 6.603), 2—right superior occipital gyrus (BA18, *t* = 6.7611), 3—left middle occipital gyrus (BA19, *t* = 6.4052), and 4—right postcentral gyrus (BA2, *t* = 6.9664). Spot size denotes the degree of quantitative changes. ReHo, regional homogeneity; TA, toothache; HC, healthy control.

The results of this study showed significant increases in ReHo values in the RLG, RSOG, LMOG, and RPG (Figure 4), indicating that the stimulation caused by TA affects the above four cerebral areas.

The RLG is associated with emotional control and cognitive function. One study showed that the normal functioning of the lingual gyrus (LG) helped fight anxiety and depression and maintain normal cognitive function (Jung et al., 2014). Related studies further showed that in Alzheimer’s disease and depression, functional connectivity between LG and other cortical regions was decreased (Liu et al., 2017). Our study did not measure functional connectivity but we found that LG activity was increased in TA. Previous reports have indicated that TA experiences are associated with aversion emotions. Activation of the patient’s brain in disgust might mean that the individual was positively responding to painful stimuli, constantly reassessing the pain (Borsook et al., 2013; Baliki and Vania Apkarian, 2015). When the TA experience was related to aversion, activated brain areas included the frontal lobe, amygdala, hippocampus, and others (Duerden and Albanese, 2013; Lin et al., 2013b). These areas associated with TA have been described as the “cognitive-emotional” network, indicating that cognition and emotion play significant roles in the development of TA (Wiech et al., 2008). The main function of the occipital lobe is to process visual information. Furthermore, the RSOG responds preferentially to visual information about spatial characteristics (Renier et al., 2010). In addition, epidemiological studies have confirmed that patients with visual impairment have relatively poor periodontal health, and the incidence of gingival bleeding and dental calculus is higher than that of normal subjects, which increases the risk of toothache (Liu et al., 2019). However, the effect of toothache on visual dysfunction needs further study. On the other hand, other studies found a potential link between the lingual region of the occipital lobe and the hippocampus and amygdala, with a major change in mood due to TA associated with occipital gyrus activation (Isenberg et al., 1999; Cho et al., 2012). A related study in children also suggested that the SOG played a role in pediatric bipolar disorder (Wei et al., 2018). In addition, a resting-state imaging study showed that the functional state of the LMOG was associated with major depressive disorder in females (Teng et al., 2018). The alterations in RLG, RSOG, and LMOG activity in the present study can partially confirm these findings. The functional state of ReHo values in these three cerebral cortex regions was related to the body’s emotional and cognitive functions, and changes in their ReHo values may affect their functions.

Patients with TA often experience unbearable pain. It has been found that pain activates the primary (S1) and secondary (S2) somatosensory cortex, insular cortex, cingulate cortex, and parietal and thalamus regions. Some previous studies have suggested that many pain-related diseases activate the S1 region (Peyron et al., 2000; Apkarian et al., 2005; Frot et al., 2013). Gerstner et al. (2013) found by comparing harmful stimulation with a resting baseline that the somatosensory cortex, parietal lobe, and cingulate gyrus played key roles in oral pain. The right postcentral gyrus is part of the somatosensory cortex. Integrated stimulation of the somatosensory cortex, insula, and anterior central cingulate cortex may regulate the development of TA, and our experimental results can to some extent confirm this. We, therefore, consider that change in activity at the right postcentral gyrus was due to the stimulation of the brain caused by the TA itself. Table 4 shows the functions of the above cerebral regions and the diseases associated with dysfunction.

**TABLE 4:** Brain regions alternation and its potential impact.

Brain regions	Experimental result	Brain function	Anticipated results
Right lingual gyrus	TA > HCs	Emotional control and cognitive function	Anxiety and depression
Right superior occipital gyrus	TA > HCs	Processing language, motion perception, abstract concepts, and visual information	Motor perception and visual impairment
Left middle occipital gyrus	TA > HCs	Visual information processing of spatial characteristics	Visual impairment, depression and anxiety
Right postcentral gyrus	TA > HCs	Body somatosensory	Left somatosensory disorder

HCs, healthy controls; TA, toothache.

ROC curves can be used to distinguish between healthy and diseased states and higher AUC values (above 0.7) indicate higher diagnostic accuracy. In our research, the AUC values of each cortical region were all greater than 0.9, indicating good accuracy and suggesting that ReHo at the brain regions discussed above may be a practical method for the diagnosis of TA in the future. The present study had some limitations. First, the sample is small, limiting the reliability of the experimental results (Button et al., 2013). Second, there is a lack of standardized noxious stimulation in the field of pain research, so while many clinical factors can cause TA in patients, we could not evaluate these in a standardized manner. Third, the psychological characteristics of patients with TA vary, and we could not determine the connection between these characteristics and those related to brain activity.

In summary, using ReHo to study fMRI data was an effective way to study brain regional changes in a neural functional state in TA patients. Our results showed that areas of the brain in TA patients, including the somatosensory area, exhibited altered synchronous neural activity. These results provide a basis for further understanding of TA and relevant brain mechanisms and the development of customized pain control for individual dental patients.

## Data availability statement

The original data can be obtained by contacting the corresponding author. Requests to access these datasets should be directed to Y-LH, 173386424@qq.com.

## Ethics statement

This study was reviewed and approved by the Medical Ethics Committee of the first affiliated Hospital of Nanchang University (cdyfy2017021). All procedures performed in studies involving human participants were following the 1964 Helsinki declaration and its later amendments or comparable ethical standards.

## Author contributions

JY and Y-LH contributed to the study inception and design. JY and YS equally contributed to the literature search, analysis, and writing of the manuscript. BiaL, BinL, Q-MG, Q-YL, Q-YY, R-BL, S-NW, and Y-CP contributed to the study design and study supervision. All authors approved the final version of the manuscript.
